# Regional differences in incidence and clinical presentation of type 1 diabetes in children aged under 15 years in Croatia

**DOI:** 10.3325/cmj.2012.53.141

**Published:** 2012-04

**Authors:** Gordana Stipančić, Lavinia La Grasta Sabolić, Marija Požgaj Šepec, Ana Radica, Veselin Skrabić, Srećko Severinski, Mirjana Kujundžić Tiljak

**Affiliations:** 1 Department of Pediatrics, University Hospital Center Sestre Milosrdnice, Zagreb, Croatia; 2 Department of Pediatrics, University Hospital Center Zagreb, Zagreb, Croatia; 3 Department of Pediatrics, University Hospital Center Split, Split, Croatia; 4 Department of Pediatrics, University Hospital Center Rijeka, Rijeka, Croatia; 5 Department of Medical Statistics, Epidemiology and Medical Informatics, Andrija Štampar School of Public Health, Zagreb, Croatia

## Abstract

**Aim:**

To determine regional differences in the incidence, incidence trends, and clinical presentation of type 1 diabetes in children under the age of 15 years in Croatia in a 9-year period (1995-2003).

**Methods:**

We included the patients who had been diagnosed with the disease and had started the insulin treatment before they were 15 years old. Regional differences between eastern, central, and southern Croatia were observed. The gross incidence was expressed by the number of newly diagnosed type 1 diabetes patients in 100 000 children of the same age and sex per year, ie, for the 0-14 age group, and for the 0-4, 5-9, and 10-14 subgroups.

**Results:**

The highest incidence was observed in southern Croatia (10.91 per 100 000/y) and the lowest in central Croatia (8.64 per 100 000/y), and in eastern Croatia the incidence was 8.93 per 100 000/y. All three regions showed a growing incidence trend, which was significant only in eastern and southern Croatia. There was 35.9% of patients with diabetic ketoacidosis in eastern Croatia, 41.7% in central Croatia, and 31.3% in southern Croatia.

**Conclusion:**

Croatian regions show differences in the incidence, incidence trends, and disease presentation of type 1 diabetes. A further follow-up is needed to establish whether the regional differences are a consequence of the population dynamics in the observed period or they will continue to exist, pointing to differences in environmental risk factors.

The incidence of type 1 diabetes is highest in Finland, amounting to 40.9/100 000/y, and lowest in China and Venezuela, amounting to 0.1/100 000/y (1). It varies up to by 10 times among European countries, and as much as by 400 times globally (2). These variations are mainly caused by differences in the genetic makeup of specific ethnic groups and diverse environmental factors (3,4).

Sometimes countries of a certain region have similar incidence patterns despite their genetic and long-standing socio-economic differences. A good example are Hungary (7.87/100 000/y), Austria (9.5/100 000/y), the Czech Republic (9.8/100 000/y), and Slovakia (9.2/100 000/y) (5,6). In contrast to this, certain bordering countries sharing the same genetic pool show considerable differences in their incidence rates, ie, Spain and Portugal, and Finland and the Russian province Karelia (7,8). Such cases have not only been recorded in Europe, but also in America. While the incidence for Puerto Rico is the same as for the majority of the US states (17/100 000/y), the neighboring Cuba has a considerably lower incidence, with fewer than 3 patients per 100 000/y (9).

Differences in the incidence rates have been recorded even among the regions of the same country (5,10-14). In some cases, this may be explained by the presence of a certain ethnic minority (14) with a different genetic base than the majority population. However, variations are sometimes noted in genetically more homogeneous populations, which points to environmental factors as the possible cause of the differences (10,11). Some studies have shown that changes in the incidence in different regions do not necessarily follow the same pattern over a course of time (15).

Establishing the regional distribution of a disease is an important epidemiological method, which may lead to certain etiological hypotheses (10). Since the national incidence and clinical presentation patterns of type 1 diabetes in Croatia had already been established (16,17), the aim of this study was to determine regional differences in the incidence, incidence trends, and clinical presentation of type 1 diabetes in children under the age of 15 years within a 9-year period.

## Materials and methods

In Croatia, children diagnosed with or suspected to have diabetes start treatment in hospital pediatric departments. Hospital pediatricians experienced in the care of diabetic children establish the definitive diagnosis of the disease, introduce therapy, educate both parents and children, and ensure adequate follow-up. Therefore, the primary data source for this study was information obtained by direct contact in writing with the hospital pediatricians treating diabetic children. All 18 hospitals in Croatia where the treatment of children with diabetes is performed were included in data collection. Capture-recapture method (18,19) and a secondary data source estimated the number of possibly omitted patients.

Data for the period from 1995 to 1998 were collected retrospectively, and those for the period from 1999 to 2003 prospectively. At the end of each calendar year, all the involved pediatric departments provided completed questionnaires containing information on newly diagnosed patients, including their names, dates of birth, sex, first dosing dates, and addresses (web-extra material) (web extra material 1). The questionnaire also contained additional questions about symptoms prior to diagnosis and their duration, the level of metabolic disorder at the time of diagnosis, as well as the history of diabetes in first-degree relatives. Data concerning the clinical presentation at the onset of the disease were collected retrospectively from available case histories for the whole group.

Blood glucose concentrations, pH, and bicarbonate values were obtained by measuring the glycemic and acid-base status (ABS), which are routine procedures for determining the level of metabolic disorder in newly diagnosed patients. Blood glucose concentrations were measured by the standard glucose-oxidase method. The highest value measured within the previous 24 hours was considered before introducing insulin therapy. pH and bicarbonate values were obtained by determining ABS at hospital admittance. Diabetic ketoacidosis was defined either as 1) pH<7.3 or 2) pH<7.3 and/or bicarbonates <15 mmol/L.

The patients involved in the study were diagnosed with type 1 diabetes between January 1, 1995 and December 31, 2003. In these patients, the disease had been diagnosed and insulin treatment had been started before they reached 15 years. The children were residents of Croatia for a minimum of 6 months before the diagnosis. The diagnosis of type 1 diabetes was established according to the WHO criteria (20). The day of the first insulin dose was taken as the point of onset of the disease.

Information on the membership in the Croatian Diabetes Association served as a secondary data source. Members who started the insulin therapy before the age of fifteen and were registered in the period from January 1, 1995 to December 31, 2003 were filtered out of the database.

**Figure 1:**
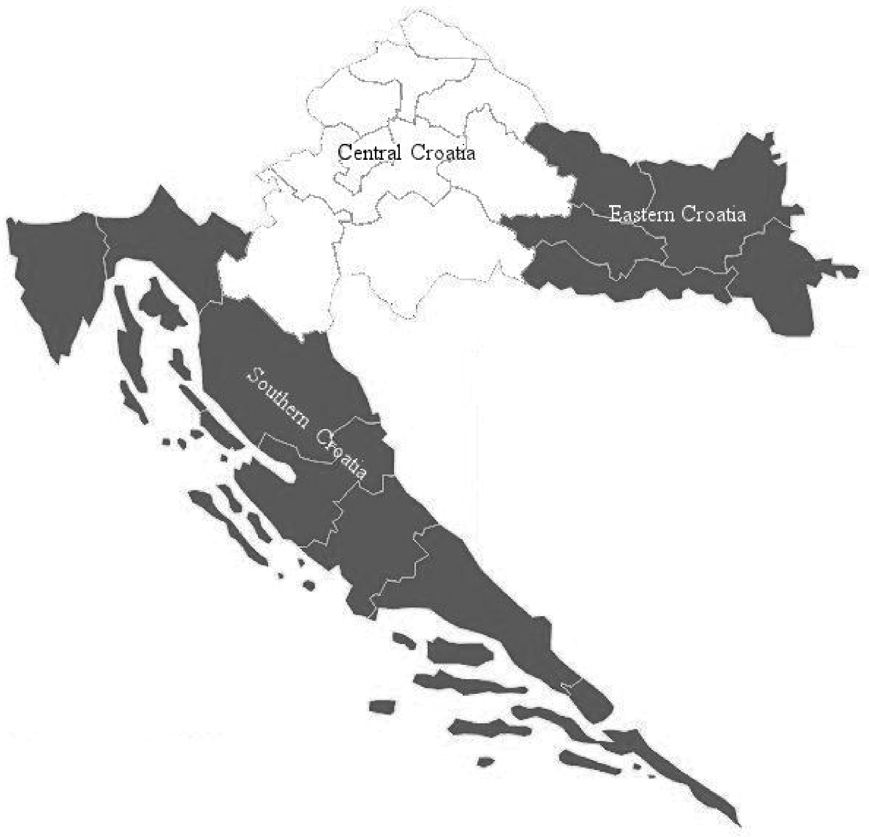
The observed Croatian regions (eastern, central, and southern Croatia).

We observed regional differences among three areas: eastern, central, and southern Croatia on a county basis (Figure 1). Eastern Croatia comprises Osječko-baranjska, Vukovarsko-srijemska, Brodsko-posavska, Požeško-slavonska, and Virovitičko-podravska county. Central Croatia, the northwestern and the most densely populated part of the country including almost half of the country’s population comprises the following counties: Zagrebačka, Krapinsko-zagorska, Varaždinska, Međimurska, Koprivničko-križevačka, Bjelovarsko-Bilogorska, Sisačko-Moslavačka, Karlovačka, and the city of Zagreb. Southern Croatia comprises the mountainous part in the southwest, Istria and the Primorje region in the Adriatic north, as well as the southernmost region of Dalmatia, ie, seven counties: Primorsko-goranska, Istarska, Ličko-senjska, Zadarska, Šibensko-kninska, Splitsko-dalmatinska, and Dubrovačko-neretvanska. Ethical approval was obtained from the Sestre Milosrdnice University Hospital Center Ethics Committee.

### Statistical analysis

The data reliability regarding newly diagnosed patients was tested by the capture-recapture method, which uses two independent data sources (18,19). The gross incidence was expressed by the number of newly diagnosed type 1 diabetes patients per 100 000 children of the same age and sex per year, ie, for the 0-14 age group as well as for the 0-4, 5-9, and 10-14 subgroups, in total, and according to sex. The population data were obtained from the Croatian Bureau of Statistics, based on the 1991 and 2001 censuses, as well as on the annual estimates for the periods between the censuses. The number of children aged 0-14 decreased from 921 186 in 1995 to 729 300 in 2003. Data on specific regions were obtained from the estimates for 2001, 2002, and 2003, and from the estimates for 2001 for 1995 to 2000 (21).

95% confidence intervals (CI) were estimated by the Wald method with the application of the normal approximation of the Poisson distribution (22,23). For the calculation of standardized incidence, the direct method was applied on the standard global population for the 0-14 age group, in total, and according to sex. The logistic regression method was used to analyze the significance of regional differences associated with ketoacidosis incidence.

The Poisson regression model was applied for the analysis of incidence changes in the period from 1995 to 2003, incidence trend changes among the age groups, and between the sexes, and for the comparison of the groups according to regional differences. *P* values lower than 0.05 were considered statistically significant. Data were processed using SAS (SAS Institute, Cary, NC, USA) and STATISTICA (StatSoft, Tulsa, OK, USA) software (both licensed by the University of Zagreb, Zagreb, Croatia).

## Results

### Determining the number of patients and incidence for the whole territory of Croatia

There were 692 children (323 girls and 369 boys) diagnosed with type 1 diabetes. The lowest number of patients (134) was found in the youngest age group (0-4 y), and the number increased with age (255 in the 5-9 y group, 303 in the 10-14 y group).

From 1995 to 1998, out of 273 diagnosed children, data for 271 were obtained from the primary source (hospital records), for 2 from the secondary source (the Croatian Diabetes Association records), and for 120 from both sources. The estimate reached by the capture-recapture method for the total number of newly diagnosed patients was 275, with data reliability of 98.5%. From 1999 to 2003, out of 419 diagnosed children, data for 147 were obtained from both sources, for 414 from the primary source, and for 5 only from the secondary source. The estimate reached by the capture-recapture method for the total number of newly diagnosed patients was 428, with data reliability of 96.7%. Data reliability for the entire period from 1995 to 2003 was 97.4%. The two observed periods did not differ significantly in data reliability (*P* = 0.057).

Data on type 1 diabetes incidence for the whole Croatia had already been published, and we used them for the comparison of incidence and disease presentation in whole Croatia and different regions (16,17).

### Incidence of type 1 diabetes according to region

Table 1 shows the number of diagnosed children and the prevalence by sex and age for each region. It also presents the incidence rates in different Croatian regions for the 0-14 age group, for both sexes, and according to sex, as well as for the 0-4, 5-9, and 10-14 age subgroups.

**Table 1:** Type 1 diabetes incidence in children – the gross incidence rate per 100 000 person/y in Croatia and different Croatian regions from 1995 to 2003

Age/sex (years)	Croatia*			Eastern Croatia			Central Croatia			Southern Croatia		
	number of patients	incidence rate	95% confidence interval	number of patients	incidence rate	95% confidence interval	number of patients	incidence rate	95% confidence interval	number of patients	incidence rate	95% confidence interval
**0-4**	**134**	**5.8**	**4.8-6.7**	**38**	**7.6**	**5.4-10.8**	**57**	**5.6**	**4.2-7.4**	**40**	**6.0**	**4.3-8.3**
**5-9**	**255**	**9.8**	**8.6-11.0**	**47**	**9.1**	**6.7-12.3**	**107**	**10.3**	**8.4-12.6**	**101**	**14.1**	**11.5-17.4**
**10-14**	**303**	**11.1**	**9.9-12.4**	**55**	**10.3**	**7.7-13.6**	**125**	**11.2**	**9.3-13.5**	**122**	**15.4**	**12.7-18.6**
**0-14**	**692**	**9.1**	**8.4-9.7**	**140**	**8.9**	**7.4-10.7**	**289**	**8.6**	**7.6-9.8**	**263**	**10.9**	**9.4-12.6**
**0-14 boys**	**369**	**9.4**	**8.5-10.4**	**74**	**9.5**	**7.4-12.1**	**155**	**9.3**	**7.8-11.0**	**140**	**11.8**	**9.8-14.2**
**0-14 girls**	**323**	**8.7**	**7.7-9.6**	**66**	**8.4**	**6.5-10.9**	**134**	**8.0**	**6.7-9.7**	**123**	**10.1**	**8.2-12.4**

*Data for Croatia as a whole are obtained from previous studies (16,17).

The highest annual incidence growth was observed in eastern Croatia. The incidence in the 0-14 years age group grew from 3.9 per 100 000/y (95% CI, 1.6-9.2) in 1995 to 15.7 per 100 000/y (95% CI, 10.0-24.4) in 2003. During the entire observed period, a significantly rising incidence trend was recorded and a significant incidence growth was observed in all age subgroups. The biggest annual incidence growth was observed in the youngest age subgroup (0-4 y) (Table 2).

**Table 2:** Incidence trend for type 1 diabetes overall and according to age groups in different Croatian regions from 1995 to 2003.

Age/y	Croatia*			Eastern Croatia			Central Croatia			Southern Croatia		
	annual incidence increase (%)	95% confidence interval	*P*	annual incidence increase (%)	95% confidence interval	*P*	annual incidence increase (%)	95% confidence interval	*P*	annual incidence increase (%)	95% confidence interval	*P*
**0-4**	**14.0**	**8.8-22.5**	**<0.001**	**16.0**	**6.1-27.1**	**<0.000**	**8.9**	**0.6-17.9**	**0.030**	**11.7**	**2.7-21.4**	**0.009**
**5-9**	**8.3**	**3.2-13.7**	**<0.001**	**9.2**	**0.7-18.4**	**0.030**	**2.5**	**0.0-9.0**	**0.420**	**5.1**	**0.0-11.9**	**0.120**
**10-14**	**7.7**	**3.1-12.7**	**<0.001**	**9.1**	**0.9-18.0**	**0.020**	**2.4**	**0.0-8.6**	**0.410**	**5.0**	**0.0-11.5**	**0.100**
**0-14**	**9.0**	**5.8-12.2**	**<0.001**	**11.4**	**3.9-19.4**	**0.002**	**4.6**	**0.0-9.8**	**0.060**	**7.2**	**1.8-12.9**	**<0.008**

*Data for Croatia as a whole are obtained from previous studies (16,17).

The lowest incidence was recorded in central Croatia. There was an incidence rise from 9.1 per 100 000/y (95% CI, 6.1-13.6) in 1995 to 12.8 per 100 000/y (95% CI, 9.2-18.0) in 2003. Although during the entire observed period a rising incidence trend was recorded, it was not significant. A significant incidence growth was observed only in the youngest (0-4 y) age subgroup, while there was no significant incidence growth in the oldest age subgroups (Table 2).

The highest incidence was recorded in southern Croatia, with incidence growth from 10.2 per 100 000/y (95% CI, 6.5-16.0) in 1995 to 15.3 per 100 000/y (95% CI, 10.5-22.3) in 2003. During the entire observed period, a significantly rising incidence trend was recorded. However, comparing the age subgroups, the incidence growth was significant only in the youngest age subgroup (0-4 y). In the older age subgroups, the annual incidence growth was lower and not significant (Table 2).

In all regions, the incidence was lowest in the youngest age subgroup (0-4 y) and was growing proportionally with age (Table 1). The boy/girl ratio was almost the same in all three regions (eastern 1.12, central 1.15, southern 1.13).

### Comparison among regions

Poisson regression analysis showed that while there were differences among the regions in the incidence (*P* = 0.004), there was no difference in the incidence trend (*P* = 0.286). (Table 3). A difference in incidence was observed between central and southern Croatia (*P* = 0.017), while there were no differences between either eastern and central Croatia (*P* = 0.770) or between eastern and southern Croatia (*P* = 0.080). There were also no differences in the incidence among the age subgroups in the various regions (*P* = 0.186). Although the incidence among boys was higher than among girls in all three regions, sex was not a significant factor influencing the incidence in either region (*P* = 0.994). (Table 3)

**Table 3:** Poisson regression analysis showing differences in incidence of type 1 diabetes and impact of sex and age groups on incidence in the Croatian regions from 1995 to 2003

Source	DF*	Χ^2^	P
**Region**	2	11.03	0.004
**Year, region^†^**	2	2.50	0.286
**Sex, region^‡^**	2	0.04	0.999
**Age groups, region**^§^	4	6.19	0.186

*Degrees of freedom.

†Interaction between trend across the nine years and region.

‡Interaction between sex and region.

§Interaction between age groups (0-4y, 5-9y, 10-14y) and region.

*Regional differences in the incidence of diabetic ketoacidosis*. Data pertaining to the clinical presentation at diagnosis were collected from 607 out of 692 patients (87.7%), 289 girls and 318 boys. Mean age at diagnosis was 8.79 years, ranging from 0.2 to 14.9 years. There were 121 patients younger than 5 years, 228 aged 5-9 years, and 258 aged 10-14 years. At disease onset, more than one third of patients presented with diabetic ketoacidosis defined as pH<7.3 (Table 4).

**Table 4:** Incidence of diabetic ketoacidosis in different Croatian regions from 1995 to 2003.

	No. (%) of participants from			
	Eastern Croatia	Central Croatia	Southern Croatia	Croatia
**pH≥7.3**	75 (64.1)	144 (58.3)	167 (68.7)	386 (63.6)
**pH<7.3**	42 (35.9)	103 (41.7)	76 (31.3)	221 (36.4)
**All**	117 (19.3)	247 (40.7)	243 (40.0)	607 (100.0)

*Data for Croatia as a whole are obtained from previous studies (16,17).

Logistic regression analysis indicated that regional differences in diabetic ketoacidosis prevalence were significant (*P* = 0.029). A significant difference was established between central and southern Croatia (*P* = 0.036). In central Croatia, the disease was 1.68 times more likely to present with diabetic ketoacidosis than in southern Croatia (95% CI, 1.1-2.5). There was no significant difference between either eastern and central Croatia (*P* = 0.310) or between eastern and southern Croatia (*P* = 0.937). Body mass index (BMI) Z-score was calculated from body weight and height at diagnosis. Mean BMI Z-score was -0.6 ± 1.4 (from -3.9 to 3.4). There were no regional differences in BMI Z-scores (F = 0.71, *P* = 0.494).

## Discussion

This study found significant regional differences in the incidence of type 1 diabetes in children in Croatia. The highest incidence was observed in southern Croatia, and it was significantly higher than in central Croatia. There were no regional differences in incidences according to age groups. Although the incidence was higher among boys in all regions, the regional differences in incidence according to sex were not significant.

Other countries have significant regional differences in the incidence of type 1 diabetes. In Sardinia, the two studies conducted in the period from 1989-1999 have shown the incidence growth but without regional differences in this genetically homogeneous population (24,25). However, the incidence of type 1 diabetes in Sardinia significantly exceeds the rest of Italy. Regional differences were also observed in the continental part of Italy (12).

The western part of genetically and ethnically homogeneous Hungary had a 1.3 times higher incidence than the central and eastern part (5). Romania, a country with a low incidence, had a high variability of up to 6.7 times among its regions (13). Similar variations have also been recorded in non-European countries, eg, in New Zealand, where the incidence is 1.5 times higher in the south than in the north. This difference is clearly related to the prevalence of Caucasians of European origin in the south and the Maori ethnic group in the north (14).

The considerable variance in the incidence of type 1 diabetes not only between countries but also between regions within a country points to a conclusion that the etiological factors of this disease are rather complex, and that the role of environmental factors is as important as that of the genetic ones. Regarding genetic factors, it appears that incidence variations are mainly affected by the existence of a special DQ genotype (HLA-DQ2/DQ8 and HLA-DQ4/DQ8) in the population, whereas environmental factors are numerous and complex, involving climatic, infectious, nutritional, and even socio-economic (26). The analysis of regional differences in the incidence of a particular disease is a key feature in the planning of further research into the definite causative factors (24).

In the course of the nine-year study on the entire territory of Croatia, a growing incidence trend was observed with an annual rise of 9% (16). Although a growing incidence trend was recorded in all the three observed regions in our study, the growth was significant only for eastern and southern Croatia, but not for central Croatia. Eastern Croatia led with an annual incidence growth rate of 11.4%, while the growth rates in southern and central Croatia were lower (7.2% and 4.6%, respectively). It should also be noted that in all the regions a significantly rising incidence trend was present in the youngest age group, while the trend significance for the older age groups (5-9 y and 10-14 y) disappeared for central and southern Croatia.

Analyses of regional differences are complemented by ecological analyses aiming to establish factors contributing to the incidence of a particular disease. The features that are usually considered are differences between urban and rural environments, population density, representation of children in the population, population migrations, isolation of the area, socio-economic characteristics, etc. In addition, potential polluting agents, dietary features, as well as population nourishment and other factors may be analyzed. Regarding type 1 diabetes, most studies point to higher incidences in rural, isolated, and less densely populated regions with a lower migration rate (10,27) and lower incidences in urban areas (28). These observations might be in accordance with the “hygiene theory,” which states that the proper development of the immune system is affected by an early and sufficient exposition to infectious agents. Otherwise, the insufficiently developed system will react inadequately to future stimulants, leading to the development of autoimmune diseases, like type 1 diabetes (29). In urban environments, due to frequent social contacts children are exposed to infection earlier and more frequently, which may have a future protective role in the incidence of type 1 diabetes. The same explanation may be applicable for the higher incidence of the disease in less densely populated or isolated areas, as well as in areas of lower migration and consequentially lower population mixing rates.

In order to explain the regional incidence differences observed in this study, it must be mentioned that the results pertain to a very dynamic period for our country; due to the war, extensive population migrations occurred especially at the regional level. For comparison of regional incidences, the population estimates for 2001, 2002, and 2003 were used. The population estimates for these years were considerably closer to the situation in 1995 and after than to the 1991 census (21). Therefore, the results should be interpreted with caution, taking into account that differences exist, but that a continued follow-up is necessary in order to confirm them. The lowest incidence rate in central Croatia may be associated with the fact that it includes the largest urban settlement, the city of Zagreb, besides being an area affected by significant population migrations. However, eastern Croatia with its even higher migration rates had the largest incidence growth. BMI, whose values might be associated proportionally with a higher incidence of the disease, were not a factor affecting the regional incidence differences.

This study pointed out significant regional differences in the disease presentation. The highest prevalence of diabetic ketoacidosis at diagnosis was in central Croatia, significantly higher than in southern Croatia. However, the incidence of the disease was significantly higher in southern than in central Croatia. It is assumed that incidence inversely correlates with the prevalence of diabetic ketoacidosis at diagnosis; thus countries with a higher incidence have a smaller proportion of patients presenting with diabetic ketoacidosis at diagnosis. This can be attributed to a higher awareness and greater knowledge about the disease, with the ability of its early recognition (30,31). It is often emphasized that countries with a high incidence of type 1 diabetes are also those with a higher standard of living, which entails better health care and management of children with type 1 diabetes. Consequently, this may be the reason for a lower prevalence of diabetic ketoacidosis at diagnosis (32).

A further follow-up is needed to establish whether the regional differences were a consequence of the population dynamics within the observed period or whether they will continue to exist and thereby point to differences in environmental factors.
