# Supplementary material for: Regional differences in incidence and clinical presentation of type 1 diabetes in children aged under 15 years in Croatia
Source: Croat Med J. 2012 Apr;53(2):141–8. doi: 10.3325/cmj.2012.53.141 (PMC3342644; doi:10.3325/cmj.2012.53.141)
Supplement: Supplementary Table 1 [file CroatMedJ_53_s001.pdf]

Incidence and clinical presentation of type 1 diabetes in children aged 0-14 years in Croatia, period 1995-2003

First Name and Surname, date of birth, gender<br>Address	
Date of first insulin administration	
Symptoms: polyuria<br>nicturia<br>enuresis<br>polydipsia<br>weight loss<br>others	YES NO<br>YES NO<br>YES NO<br>YES NO<br>YES NO
Duration of symptoms (in days or weeks)	
Infection: before symptoms' onset<br>simultaneous with the symptoms	YES NO<br>YES NO
First hospital admission: weight (kg)<br>height (cm)	
GUK (mmol/l)	
Acide-base status: pH<br>bicarbonate (mmol/l)	
HbA1C %	
Diseased family member: mother<br>father<br>brother<br>sister	YES/type NO<br>YES/type NO<br>YES/type NO<br>YES/type NO
